# Identification of SYNJ1 in a Complex Case of Juvenile Parkinsonism Using a Multiomics Approach

**DOI:** 10.3390/ijms25179754

**Published:** 2024-09-09

**Authors:** Ester Leno-Durán, Luisa Arrabal, Susana Roldán, Inmaculada Medina, Clara Alcántara-Domínguez, Victor García-Cabrera, Jorge Saiz, Coral Barbas, Maria José Sánchez, Carmen Entrala-Bernal, Francisco Fernández-Rosado, Jose Antonio Lorente, Purificacion Gutierrez-Ríos, Luis Javier Martínez-Gonzalez

**Affiliations:** 1 Department of Obstetrics and Gynecology, Faculty of Medicine, University of Granada, 18016 Granada, Spain; 2 Pediatric Neurology Department, Hospital Virgen de las Nieves, 18014 Granada, Spain; luisillaarrabal@hotmail.com (L.A.); sroldanaparicio@gmail.com (S.R.); inmamed@hotmail.com (I.M.); 3 Centre for Genomics and Oncological Research (GENYO), Pfizer, University of Granada, Andalusian Regional Government, PTS, 18016 Granada, Spain; clara.alcantara@genyo.es (C.A.-D.); victor.garcia@genyo.es (V.G.-C.); jose.lorente@genyo.es (J.A.L.); luisjavier.martinez@genyo.es (L.J.M.-G.); 4 Centre for Metabolomics and Bionanalysis (CEMBIO), Chemistry and Biochemistry Department, Pharmacy Faculty, Universidad CEU San Pablo, 28926 Madrid, Spaincbarbas@ceu.es (C.B.); 5 CIBER Epidemiology and Public Health (CIBERESP), 28029 Madrid, Spain; mariajose.sanchez.easp@juntadeandalucia.es; 6 Andalusian School of Public Health (EASP), 18080 Granada, Spain; 7 Instituto de Investigación Biosanitaria, ibs. GRANADA, 18012 Granada, Spain; 8 Lorgen G.P., PT, Ciencias de la Salud-Business Innovation Centre (BIC), 18016 Granada, Spain; carmen@lorgen.com (C.E.-B.); javier@lorgen.com (F.F.-R.); 9 Laboratory of Genetic Identification, Legal Medicine and Toxicology Department, Faculty of Medicine-PTS, University of Granada, 18016 Granada, Spain; 10 Jaen-Sur Health District, Andalusian Health Service, Andalusian Regional Government, 23009 Jaén, Spain; 11 Department of Biochemistry, Molecular Biology III and Immunology, Faculty of Medicine, University of Granada, 18016 Granada, Spain

**Keywords:** Juvenile Parkinsonism, SYNJ1, whole-exome sequencing, mutation screening, metabolism, multiomics

## Abstract

This study aimed to elucidate the genetic causes underlying the juvenile parkinsonism (JP) diagnosed in a girl with several family members diagnosed with spinocerebellar ataxia type 2 (SCA2). To achieve this, whole-exome sequencing, analysis of CAG repeats, RNA sequencing analysis on fibroblasts, and metabolite identification were performed. As a result, a homozygous missense mutation SNP T>C (rs2254562) in synaptojamin 1 (SYNJ1), which has been implicated in the regulation of membrane trafficking in the synaptic vesicles, was identified. Additionally, we observed overexpression of L1 cell adhesion molecule (L1CAM), Cdc37, GPX1, and GPX4 and lower expression of ceruloplasmin in the patient compared to the control. We also found changes in sphingolipid, inositol, and inositol phosphate metabolism. These findings help to clarify the mechanisms of JP and suggest that the etiology of JP in the patient may be multifactorial. This is the first report of the rs2254562 mutation in the SYNJ gene identified in a JP patient with seizures and cognitive impairment.

## 1. Introduction

Parkinson’s disease (PD) is a progressive neurodegenerative disorder of the central nervous system with multifactorial etiology, and it is estimated to affect approximately 2% of people over 60 years old. The pathogenesis of PD is not fully understood. While 5–10% of patients have a family history of PD, the majority of the cases are idiopathic and are thought to result from a complex interplay of genetic, epigenetic, and environmental factors.

The hallmark clinical features of parkinsonism include movement disorder, such as resting tremor, muscle stiffness, bradykinesia, postural instability, and gait disturbance. In addition, PD patients may also exhibit non-motor symptoms, such as cognitive impairment, olfactory dysfunction, sleeping disturbance, hyposmia, depression, behavior disorder, and autonomic dysfunction [1].

The average age of the onset of PD is 60 years; early-onset PD is defined as the onset of PD symptoms before the age of 40, whereas juvenile parkinsonism (JP) refers to the manifestation of parkinsonian symptoms before the age of 21 years [2,3,4]. Although PD is the second most frequently diagnosed neurological disorder, early-onset parkinsonism is rare, and JP is even rarer.

The clinical features of parkinsonism in infancy, childhood, or adolescence remain controversial due to the development of an immature brain, that is, they are highly heterogeneous both clinically and genetically. Heterogeneous characteristics include resting tremor, bradykinesia, and hypotonia, as well as atypical features, such as epilepsy, early cognitive decline, behavior problems, ataxia, dystonia, and spasticity [3,4]. Diagnosis can therefore be a challenging due to the extensive and complex phenotype, with multiple manifestations and changes over time. A classification system has been proposed based on factors such as age on onset, clinical symptoms, outcome, and etiological background [3].

Neuropathologically, parkinsonism, including PD, is characterized by the loss of dopaminergic neurons in the substantia nigra and the presence of Lewy bodies (LB) in the brain, accompanied by α-synuclein (α-syn) aggregation. Neurodegeneration is also associated with iron and calcium deposition in the brain as well as metabolic and mitochondrial disorders [5]. However, this pathology is not always present in all cases of parkinsonism, and it may also be present in several other neurological diseases.

In the last 20 years, advances in next-generation sequencing (NGS) have led to the identification of an increasing number of genes linked to JP. The most prevalent gene associated with autosomal dominant JP is *SNCA*, whereas *PARK2*, *PINK1*, and *DJ1* are related to autosomal recessive cases. Recently, other atypical mutations in genes, such as *ATP13A2*, DCTN1, VPS13C, PTRHD1, *PLA2G6*, *FBXO7*, *DNAJC6*, and *SYNJ1* have been associated with JP, causing more severe disease [3,6,7,8]. However, currently known mutations do not account for all JP cases. Hence, the search for additional genes contributing to the disorder is not complete.

Synaptojanin 1, encoded by *SYNJ1*, is a lipid phosphatase that regulates phosphoinositide phosphatase enzymes and is expressed in several tissues, including the brain, where it has a role in the endocytosis and recycling of synapsis vesicles, which is essential for preventing neurodegeneration [9,10]. Recently, accumulating evidence indicates that mutations and variants of the SYNJ1 gene are linked to many neurological disorders, including early-onset PD and atypical JP [8,11,12,13,14,15,16], together with ataxia and seizures [11,13,15,16]. In 2013, the same homozygous missense mutation (c.773G>A, p.Arg258Gln) in the SYNJ1 gene was described in two independent families from Italy and Iran with atypical autosomal recessive JP [11,15]. One year later, the same mutation was described in two brothers affected with JP in another Italian family [12]. In 2016, a second novel homozygous missense mutation (c.1377C>A, p.Arg459Pro) in the SYNJ1 gene was reported in a consanguineous Indian family with autosomal recessive JP [14]. A third novel mutation (c.2515C>T) in the SYNJ1 gene was identified in a patient from Iran who presented asymmetric parkinsonism and seizures at 24 years of age [16]. Another novel heterozygous mutation (p.Leu1406Phefs*42 and p.Lys1321Glu) in the SYNJ1 gene was identified in two siblings with mild JP and seizures in a Tunisian family [13]. Recently, in 2021, four mutations were detected in Algeria, Senegal, and France, expanding the mutational variety of SYNJ1-associated JP [8]. The different mutations in the SYNJ1 gene suggest that alterations in synapsis trafficking may play a role in the pathogenesis of the spectrum of synaptopathies. Although patients carrying these mutations display different phenotypes, mutations in SYNJ1 might identify a risk factor for the development of neurological diseases.

In this study, we present the case of a girl who first sought medical attention at the age of 18 months due to global developmental delay and fluctuating distal tremor. She was diagnosed with JP and seizures. Family history revealed the presence of movement disorders on both maternal and paternal sides. For instance, cases of hand tremors, epilepsy, and spinocerebellar ataxia (SCA) type 2 (SCA2) were identified among family members. Given the family history and the phenotype observed in the girl, a diagnosis of SCA2 was initially considered. The aim of this study was to identify the genetic cause underlying the diagnosis of JP and seizures. Therefore, this case required a multiomics approach involving the patient and several family members to confirm which of the different variants carried could be responsible for the patient’s phenotype. Whole-exome sequencing (WES), transcriptome analysis, and metabolite identification were performed in the patient and other available family members.

## 2. Results

### 2.1. Case Description

The patient described in this study is a girl from a non-consanguineous family. She was born from a triplet pregnancy with a birth weight of 1.535 Kg. The patient first sought medical attention at the age of 18 months because of global developmental delay and fluctuating distal tremor. She started having multifocal seizures at the age of 2.5, with up to 120 seizures per day, with an electrical status epilepticus during sleep (ESES) pattern. The seizures were eventually controlled with antiepileptic treatment. However, she developed a fluctuating movement disorder characterized by dystonia, tremor, and rigid-akinetic symptoms at the age of 7. She also experienced cognitive difficulties and behavioral alterations. 

Biochemical analysis revealed normal levels of amino acids in both blood and urine samples. In addition, the levels of lactic acid, ammonia, lactate, pyruvate, and beta-hydroxybutyrate were all within the expected range in blood samples. Organic acid in urine and sialotransferrin levels were also found to be normal. Hematological analysis showed a normal complete blood count and a normal peripheral blood smear with non-vacuolated lymphocytes. The patient’s immunoglobulin levels showed a slight decrease in IgA and normal levels of IgG and IgM. Apolipoproteins A and B were within the normal ranges. Serological tests for cytomegalovirus, toxoplasma, Epstein-Barr virus, syphilis, borrelia, and viral infections were all negative. 

Levodopa, acetazolamide, flunarizine, and immunotherapy were administered in an attempt to manage the symptoms without success. Multiple electroencephalograms (EEGs) were conducted and revealed notable peak wave activity in the posterior temporal and right occipital regions, forming an ESES pattern that resolved. 

The analysis of the cerebrospinal fluid showed normal levels of amino acids, organic acids, lactate, protein profile, cultures, biochemistry, and neurotransmitters. Furthermore, abdominal ultrasound and complete radiological examination yielded normal findings. Complete ophthalmological examinations were also normal. Posterior tibial nerve somatosensory evoked potentials were normal, indicating no involvement of the posterior spinal cord. Electromyography with voluntary contraction indicated the presence of axonal motor neuropathy without signs of myopathy. Repeated stimulation showed normal results.

**Figure 1:**
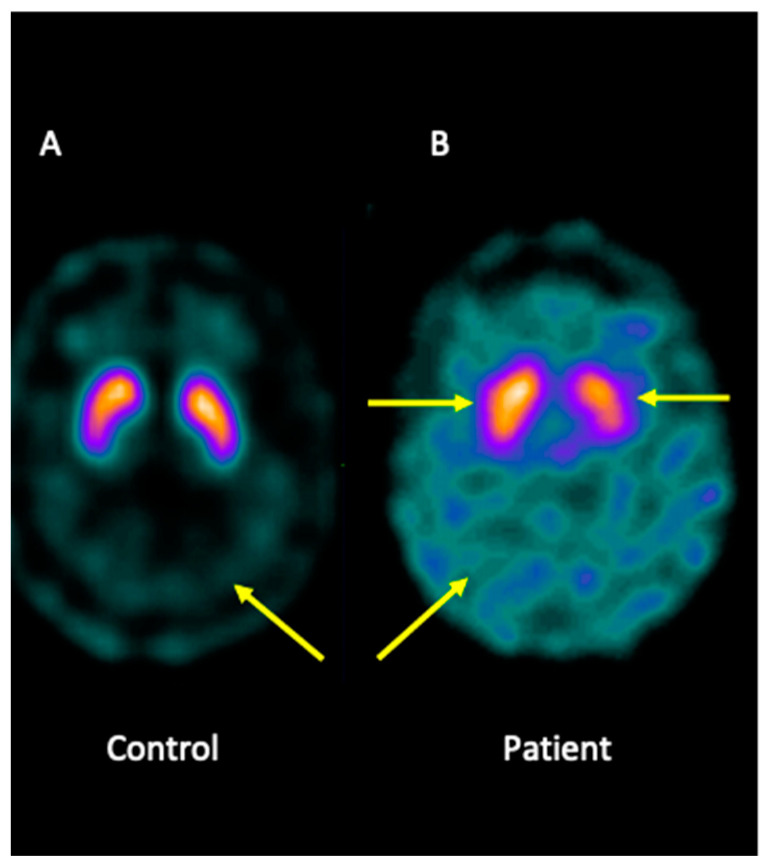
I-123-Ioflupane SPECT image of the brain patient. (**A**) Healthy control showing both striata with a normal comma shape and sharp borders, presenting low background activity. (**B**) The patient presented asymmetric striata, with a lower size in the left striata, an oval appearance, and increased background activity.

Analysis of brain imaging with SPECT using I-123-Ioflupane showed that the patient displayed asymmetric striata, with the left striata being smaller in size and oval in appearance, together with increased background activity. Furthermore, the analysis was compatible with a loss of dopaminergic activity in both striata. Overall, the pattern may be consistent with JP (Figure 1).

### 2.2. Analysis of SCA2

The father of the case was diagnosed with SCA2, an hereditary neurodegenerative disease characterized by progressive cerebellar ataxia caused by abnormal expansion of the cytosine-adenine-guanine (CAG) repeat in the ATXN2 gene. The father presented a mild phenotype, while other relatives on the paternal side, including the father’s sister and two additional female and male relatives, had more severe symptoms of SCA2 (Supplementary Figure S2). At the age of 7, the patient presented fluctuating movement disorder characterized by dystonia, tremor, and rigid-akinetic. Given the family history and the phenotype observed in the girl, an initial diagnosis of SCA2 was considered. To determine whether these signs were related to the ataxia genotype, we analyzed CAG repeat expansion not only in the patient but also in the mother and both brothers. However, no pathological genotypes were found, and the CAG repeat expansions were within the normal range (Figure S1).

### 2.3. Whole-Exome Analysis

To determine the cause of the patient’s disease, WES was performed on the patient and available family members, including both parents and both siblings. Variant filtering, such as frequency < 2% and by type, synonymous and UTR variants, and non-coding intronic variants, were used to find the genes responsible for the phenotype. Missense and loss of function variants, nonsense and frameshift mutations, splice site alterations, stop codon losses, codon insertions and deletions, and non-synonymous substitutions were selected. Several family members showed symptoms related to SCA2, including seizures, mental retardation, and early onset of cerebellar ataxia. Initial analysis of genes associated with these diseases, including SCA2, GLUT1, and ATP1A3, revealed no mutations. Regarding the gene associated with JP, the patient did not carry the main mutations known to cause JP in the PARKIN, PINK, and DJ1 genes. In addition, the patient did not present mutations in the GLUT1 and ATP1A3 genes. However, sequence analysis revealed genetic variants in three genes in the patient: SYNJ1, ACOT4, and ACAD9. 

We identified a homozygous missense mutation in the SYNJ1 gene in our proband. This mutation was a SNP T>C (rs2254562) located in exon 8 of *SYNJ1*, resulting in a missense substitution (G = Arg, A = Lys, Lys295Arg). The SYNJ1 gene is located on the long arm of chromosome 21, 21q22.11, position 32,687,042. Analysis of the patient’s family members revealed that both parents and one of the patient’s brothers carried a heterozygous missense mutation (TC) in SYNJ1. Notably, the other sibling was also found to have the same homozygous mutation, but he did not currently exhibit any neurological symptoms. The patient did not have any of the previously reported mutations in the SYNJ1 gene related to JP [8,11,12,13,14,15,16].

WES analysis identified three mutations in the patient’s ACOT4 (acyl-CoA thioesterase) gene. This gene encodes a peroxisomal enzyme involved in the hydrolysis of acyl-coenzyme A to the free fatty acid and coenzyme A [17,18,19,20]. Analysis of the patient’s ACOT4 gene showed a homozygous TCCA insertion (rs373880503). Both parents, aunt, and grandfather carried the same TCCA insertion in a heterozygous state. Additionally, a second mutation, a CTTA deletion (rs375801976), was found in this gene. Moreover, a missense variant, rs35724886 (C>A), resulting in an alanine to aspartic acid change, was also detected, although to date there is no information in the literature relating these mutations to PD [20].

Our analyses also revealed a mutation in the ACAD9 (Acyl-CoA-dehydrogenase 9) gene, which is a flavoprotein implicated in the process of beta-oxidation of unsaturated long-chain acyl-CoAs. A mutation in the ACAD9 gene, homozygous deletion GA, which has not been previously described, was found in the patient’s exome analysis. The study of this mutation in the patient’s family showed that both parents, the patient’s grandfather, and three sisters of the father carried heterozygous deletion GA in the ACAD9 gene. Mutations in ACAD9 have been associated with conditions such as cardiomyopathy, muscular weakness, and exercise intolerance, and in rare cases, patients may also present with other symptoms, such as epilepsy, intellectual deficits, and severe developmental delays [21].

### 2.4. Transcriptome Analysis

To assess the impact of the mutations identified in the patient’s genotype, we conducted next-generation transcriptome sequencing (RNA-seq) on fibroblast cell culture derived from the patient’s skin. A human foreskin fibroblast (HFF) cell line was used as a control. To identify differentially expressed genes between the patient and the control, we compared gene expression levels. We then focused on analyzing the role of the ten most overexpressed genes and the ten genes with the lowest expression in the patient compared to the control. The ten genes with the highest expression in the patient’s fibroblast cells were RPS4Y1, L1CAM, GPX1, Cdc37, GPX4, EMP3, S100A16, RPS21, PSMD8, and EDF1. The ten genes with the lowest expression in the patient compared to the control were SP9, CD160, EYS, AC09985.2, EPHA3, C4orf47, ceruloplasmin (CP), SFRP2, EMCN, and LHX8 (Table 1). Of the above 20 genes differentially expressed in the patient, 5 genes, L1CAM, Cdc37, GPX1, GPX4, and CP, warrant further investigation as they may be linked to the patient’s phenotype.

### 2.5. Metabolome Analysis

Untargeted metabolomics approaches were used to analyze changes in metabolites in plasma and urine samples. A total of 25 metabolites were identified in plasma, and 16 metabolites were identified in urine. Table 2 and Table 3 show the main percentage changes in 41 metabolites in plasma and urine, respectively. Compared with the control, levels of 11 plasma metabolites and 16 urine metabolites were increased in the patient, and levels of 14 plasma metabolites were decreased in the patient.

Plasma metabolite analysis showed that levels of several metabolites involved in sphingolipid metabolism, including O-phosohoethanolamine, sphingosine, sphingosine 1-phosphate (S1P), sphinganine 1-phosphate, and N-methylalanine were decreased in the patient. In addition, levels of myo-inositol and phosphoric acid were found to be increased in the patient, suggesting a disturbance in inositol phosphate and inositol metabolism. Furthermore, a significant increase was observed in the level of 3-carboxy-4-methyl-5-propyl-2-furanpropionic acid, a metabolite related to lipid and fatty acid metabolites, along with a slight increase in levels of phosphatidylcholines PC(18:3) and PC (20:3), and a decrease in levels of PC(18:0), PC(22:4), and arachidonic acid in the patient. With regard to phospholipid metabolism, the levels of PE (16:0), PE(18:0), and PE(20:3) were lower in the patient than in the control. Levels of uric acid, which is involved in purine metabolism, was found to be elevated in our case, while guanosine levels were decreased. Regarding amino acid metabolism, the patient had elevated levels of pipecolic acid or N-methyl-L-proline. Moreover, levels of 3’-AMP and N-Acetylneuraminic acid were slightly decreased, and those of bilirubin, 1-methylnicotinamide, and hydroxymethylbilane were increased (Table 2).

In urine analysis, we observed a high increase in the level of glucose 6-phosphate, which is related to inositol and inositol phosphate metabolism. Glucose 6-phosphate also plays a role in nucleotide sugar metabolism and is a participant in glycolysis. In addition, levels of dimethylallylpyrophosphate and isopentenyl pyrophosphate, which are involved in steroid biosynthesis, were increased in the patient. With regard to amino acid metabolism, we observed increased levels of Ne,Ne dimethyllysine, some dipeptides, and γ-glutamyl-β-aminopropiononitrile in the patient. We also found an increase in levels of metabolites related to lipid and fatty acid metabolism in the patient, including 2-hydroxymyristoylcarnitine, phosphatidylinositol PI(36:1), 3-methyladipic acid or pimelic acid, droxy-4-(methylthio) butanoic acid, dimethylallyl pyrophosphate or isopentenyl pyrophosphate, nonanoylcarnitine, and 6-keto-decanoylcarnitine. Pipecolic acid was also detected at elevated levels in the patient (Table 3).

**Figure 2:**
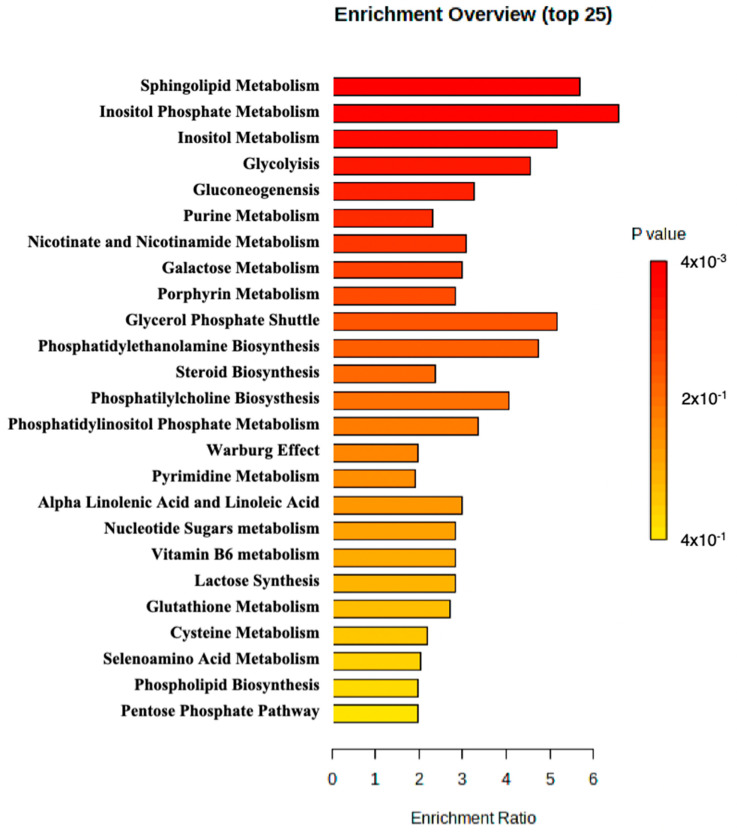
Metabolite set enrichment analysis perform by MetaboAnalyst.

MetaboAnalyst 5.0 was used to perform enrichment analysis of the available metabolite. An overview of the top 25 enriched metabolite sets is shown in Figure 2. The results suggested that sphingolipid metabolism, inositol phosphate metabolism, and inositol metabolism may be altered in the patient and potentially contribute to the pathology observed in the patient. 

## 3. Discussion

In this report, we conducted a genetic study in a patient with seizures and a suspected diagnosis of JP. Our main focus was the elucidation of the genetic etiology of JP. Several family members displayed symptoms related to SCA2, including seizures, mental retardation, and early onset of cerebellar ataxia. Initial analysis of the genes associated with these diseases, including SCA2, GLUT1, and ATP1A3, did not reveal any mutations in the patient. Hence, whole-exome sequencing was performed. We identified a homozygous missense mutation (rs2254562) in the SYNJ1 gene, three different mutations in the ACOT4 gene, and a homozygous deletion in the ACAD9 gene in the diagnosed case. We also observed overexpression of L1CAM, Cdc37, GPX1, and GPX4 in the patient compared to the control, as well as lower expression of CP. In addition, we detected alterations in sphingolipid, inositol, and inositol phosphate metabolism, suggesting their potential pathogenetic role.

To date, several different mutations in the SYNJ1 gene have been described in several families from different countries, including Iran, Italy, India, Tunisia, Algeria, Senegal, Germany, China, and France, presenting early-onset PD, atypical parkinsonism, and JP [8,11,12,13,14,15,16], suggesting a mutational hotspot. In our case, we identified a novel homozygous missense mutation SNP T>C (rs2254562) (G = Arg, A = Lys, Lys295Arg) in the SYNJ1 gene. This mutation was previously reported before by Yuam L et al. [22]. In this study, 35 variants in 22 genes related to PD, including SYNJ1 variant rs2254562, were analyzed in 512 PD patients. However, no statistically significant differences in rs2254562 were observed in that cohort between PD patients and controls in that cohort. The same research team also studied the rs2254562 variant in 200 patients with essential tremor. Again, no statistically significant differences were found in the rs2254562 variant between essential tremor patients and the controls [23]. Therefore, our report provides the first description of the rs2254562 mutation in the SYNJ1 gene in a patient with JP. 

SYNJ1 is a phosphoinositide phosphatase that regulates the levels of various phosphatidylinositols (PIs), including phosphatidylinositol 3 phosphate (PI(3)P), PI(4)P, PI(3,5)P_2_, and PI(3,4,5)P_3_, in the cellular and intracellular membranes, such as the autophagosome, endoplasmic reticulum, and Golgi apparatus [24]. Robust evidence suggests that SYNJ1 plays a role in regulating membrane trafficking in synaptic vesicles, which affects the release of neurotransmitters [9,10]. Furthermore, emerging evidence suggests its involvement in endosomal trafficking and autophagy [25,26,27]. Our analysis revealed a disruption in inositol phosphate metabolism. Altered metabolism of PIs could impact synaptic transmission and potentially affect autophagic function, thereby potentially contributing to the development of JP mediated by SYNJ1. Further research is needed to elucidate the precise mechanism of SYNJ1.

SYNJ1 is composed of three domains, two of which contain enzymatic domains crucial for lipid homeostasis and membrane trafficking: the suppressor of actin 1 (SAC1) and 5′-phosphatase, which hydrolyzes inositol species such as PI(3)P, PI(4)P, PI(3,5)P_2_, PI(4,5)P_2_, and PI(3,4,5)P_3_. The third domain is a proline-rich domain (PRD). All three domains are important in the regulation synaptic activity [26]. Mutations in both phosphatase domains of SYNJ1, R258Q, R459P, and R839C have been associated with JP or early-onset of PD, suggesting the involvement of lipid dysregulation in the pathogenesis of PD [11,12,14,28,29]. We hypothesize that the rs2254562 mutation located in exon 8 may affect the stability of SYNJ1 and its function, determining alterations in inositol metabolism, inositol phosphate metabolism, and phosphatidylinositol phosphate metabolism.

For instance, other studies have identified additional variants in *SYNJ1*, including R136*, Y888C, W843*, Q647R, and S1112T, which can lead to truncation of the protein or loss of protein expression [26,30,31].

Moreover, sphingolipids play an important role in neuron function by regulating neural growth and differentiation, mitochondrial function, autophagy, and endosomal trafficking, and these processes have been showed to be affected in PD pathogenesis [32]. For instance, S1P, a bioactive sphingolipid, acts as a neuroprotective factor by regulating neuronal cell proliferation and differentiation, stress tolerance, and protects cells from apoptosis [33,34]. Decreased plasma levels of S1P have been found in idiopathic PD, dementia with LB, multiple system atrophy, and progressive supranuclear palsy [35]. Consistent with these finding, our study showed a decrease in S1P levels in the patient compared to the control. Furthermore, in an animal model of PD, administration of fingolimod, a S1P agonist, was found to ameliorate neurodegeneration [36]. These findings suggest that abnormal sphingolipid metabolism may contribute to neurodegeneration.

Although the exact mechanisms by which SYNJ1 is involved in the pathogenesis of the disease are not fully understood, it is believed that SYNJ1 does not act alone in this process [26], and other associated factors, such as epigenetic modifications like DNA methylation or microRNAs or other molecules, may also contribute to the pathogenesis of the disease. More research is needed to elucidate the exact mechanism of SYNJ1 and the associated factors involved in the development and the progression of the disease.

Although this novel mutation in SYNJ1 may partly be responsible for the phenotype observed in this case, it is worth noting that the same mutation was found in the patient’s brother, who is currently asymptomatic. Therefore, we cannot rule out the possibility that the brother may develop the disease later in life. In fact, patients with the homozygous SYNJ1 c.773G>A mutation have developed parkinsonism symptoms in their third decade of life [8,11,12,15]. Furthermore, there is phenotypic variability and a difference in the age of onset between patients with the same mutations [11,12,13,14,15]. Another factor that may contribute to the development of the disease in the patient is the altered expression of L1CAM, Cdc37, GPX1, GPX4, and CP proteins, as well as the altered sphingolipid, inositol, and inositol phosphate metabolism observed in the patient. Based on these findings, we propose that JP observed in the patient could have multiple origins, and that the combination of features observed in this case could have led to the development of the disease.

It is well established that the accumulation and aggregation of α-syn is a major cause of the development of LB and has been implicated in the pathogenesis of PD [37,38]. Recently, it has been suggested that lipid dysregulation may play a crucial role in the abnormal aggregation of α-syn in patients with PD [39,40]. Along this line, a study conducted on postmortem PD brain samples, a cell line, and a Caenorhabditis elegans model provided evidence that the accumulation of PI(3,4,5)P_3_ leads to the aggregation of α-syn and contributes to neurodegeneration, a pathological process that has been attributed to the loss of function of a mutated SYNJ1 [41]. SYNJ1 is also associated with changes in the aggregation and phosphorylation of α-syn in mice [42].

Several studies have suggested that the spread of α-syn in extracellular vesicles in the brain may contribute to the pathogenesis of PD by affecting synaptic vesicle endocytosis [43,44,45]. Hence, high levels of α-syn have been found in extracellular L1CAM exosomes in patients with PD and atypical parkinsonism [46,47]. The L1CAM gene belongs to the immunoglobulin supergene family and encodes a protein that plays an essential role in the development of the nervous system. Neurological diseases such as hydrocephalus have been linked to mutations in L1CAM [48,49]. In addition, L1CAM exosomes containing α-syn may be involved in the pathogenesis of PD [50,51]. Our findings showed increased expression of L1CAM in our patient, indicating that it could contribute to the parkinsonism pathology due to the propagation of altered α-syn [52]. We also found overexpression of Cdc37, a co-chaperone of the cell division cycle 37, which may contribute to the phosphorylation of α-syn, affecting to its aggregation and stability [52]. It has also been reported that overexpression of Cdc37 stabilizes tau and reduces its clearance, and pathological aggregates of tau have been found in many neurological diseases, including sporadic PD [53,54]. Therefore, Cdc37 has been identified as a potential target in neurological disorders such as Alzheimer’s disease and PD [52].

Transcriptome analysis revealed overexpression of GPX1 and GPX4 in the patient compared to the control. GPX1 and GPX4 are selenoenzymes that require selenium for their antioxidant function and play an essential role in alleviating oxidative stress caused by ROS [55]. Reduced GPX activity has been reported in the serum of PD patients [56]. Overexpression of GPX enzymes such as GPX1 and GPX4 is thought to protect against neurodegenerative processes, including convulsive disorders, Alzheimer’s disease, and PD [57]. We hypothesize that increased activity of GPX1 and GPX4 was induced in response to oxidative damage, which contributed to the development of the disease in the patient. GPX1 and GPX4 are therefore attempting to compensate for an oxidative environment. This is supported by the finding that overexpression of GPX protects against neurons loss under neurotoxic conditions [58]. An experimental study using GPX knockout mice showed no neuropathological lesions. However, when the mice were injected with MPTP, a toxin that induces oxidative stress, lesions increased in homozygous GPX knockout mice, indicating that GPX is involved in antioxidant function [59]. In line with these data, GPX4 prevents neuronal death and ferroptosis by inhibiting lipid peroxidation, which has been implicated in the cell death observed in PD, ataxia, and other degenerative diseases [60,61]. Furthermore, GPX1 activity may be used as a biomarker for Friedreich’s ataxia, a fatal neurodegenerative disease [62].

Iron accumulation in the brain has been described as potential contributor to the pathogenesis of PD [63,64], and impaired iron metabolism may increase ROS levels and cause brain damage [65]. Our results showed low expression of CP, a ferroxidase enzyme that regulates brain iron homeostasis, transport, and accumulation in the brain, in the patient [66,67]. This finding suggests that CP impairment may play a role in neurodegenerative diseases characterized by oxidative damage [68,69]. Indeed, reduced serum CP levels have been identified as a risk factor for the development of PD [67]. However, no iron deposition was observed in the patient’s brain.

## 4. Materials and Methods

### 4.1. Consent and Approval

Informed consent was obtained from the adult participants and informed consent for children was obtained from the parents of all three child participants. The project received approval from the Biomedical Research Ethics Committee of the Andalusian Public Health System in Granada, Spain (1147-N-15). Sample collection and in vitro experiments were performed in accordance with ethical guidelines following the Nuremberg Code, Belmont Report, and the Declaration of Helsinki. 

### 4.2. Analysis of the Brain Imaging with Single-Photon Emission Computed Tomography 

Brain imaging analysis using single-photon emission computed tomography (SPECT) with I-123-Ioflupane was performed on the patient and a control. SPECT images were acquired using a General Electric gamma camera (Siemens Intevo-T6) and processed with Syngo MI-VA60B VA60B software to assess the image quality. The processing of the images was carried out with Workstation Syngovia VB40B-VS 06.04.0000.0040. The images were visually analyzed by a specialist.

### 4.3. Analysis of CAG Repeats in Spinocerebellar Ataxia 

Analysis of CAG repeat expansion to determine SCA2 was performed in the patient, the father, the mother, both brothers, and all of the available members of the family. DNA extraction of samples was performed using the commercial QIAamp DNA Blood Mini Kit (Qiagen). Quantification of the obtained DNA was carried out using the Qubit 2.0 fluorometer (Invitrogen, Waltham, MA, USA). Amplification of the region of interest in the ATXN2 gene was performed. Detection of the amplified fragment was performed by capillary electrophoresis under denaturing conditions in an AB PRISM^®^ 3130 Genetic Analyzer using fluorescent labeling. The results were visualized using GeneMapper 3.2.1 software.

### 4.4. WES for Candidate Gene Identification

WES analysis was conducted for the patient and all family members who were available. Genomic DNA was isolated and extracted from peripheral blood leucocytes utilizing the QIAamp DNA Blood Mini Kit (Qiagen, Hilden, Germany) and the QIAcube instrument (Qiagen, Hilden, Germany), following the manufacturer’s protocol. DNA was then fragmented to 180–220 base pair lengths using the Covaris E220 Focused-ultrasonicator (Covaris, Woburn, MA, USA). To capture exomes and prepare the library for target enrichment, 500 ng of sheared original DNA and in-solution hybridization technology were used (Roche NimbleGen SeqCap EZ MedExome; Roche Sequencing Solution, Pleasanton, CA, USA), and processing was performed using the HyperCap workflow (Roche Sequencing Solution, Pleasanton, CA, USA). MedExome design was used to obtain whole exome information, including complex regions, thereby facilitating the identification of a greater number of disease-associated variants. Then, libraries were sequences on the NextSeq 500 system (Illumina, CA, USA) using the NextSeq 500/550 High Output Kit v2.5 (300 cycles) to generate 150 × 2 paired sequences.

Reads were aligned to the human reference genome, and variant identification and and filtering were performed using the Genome Analysis Toolkit (GATK) and SAMtools mpileup pipeline. AMNOVAR and Ensembl were used for annotation, and PollyPhen-2 and SIFT were used as tools to evaluate the pathogenicity of the variants.

DNA was extracted using the DNA Blood Mini Kit (Qiagen, Hilden, Germany) by automated extraction on a Qiacube robot (Qiagen, Hilden, Germany). A total of 0.5 µg of DNA was taken from all cases and their relatives after quantification using Qubit v4 (Thermo Fisher Scientific, Waltham, MA, USA) to start the library. DNA from all samples was fragmented on the Covaris E200 (Covaris, Woburn, MA, USA) according to the Nimblegen-Roche protocol recommendations. Libraries were generated following the ‘Hybridisation-Based Target Enrichment’ protocol for exome capture using the HyperCap Workflow v2.0 and SeqCap^®^ EZ MedExome (Nimblegen Roche, Basil, Switzerland) reagents for exome library generation. 

### 4.5. RNA-Seq for Gene Expression Analysis

RNA sequencing analysis was performed on fibroblasts from both patient and control samples. The patient’s fibroblast cells were obtained via skin biopsy while fibroblasts from a human cell culture line of foreskin fibroblasts (HFF cell line) were used as a control. Cells were cultured in DMEM high glucose medium (Thermo Fisher Scientific, Waltham, MA, USA) supplemented with 10% fetal bovine serum and glutamine. RNA was isolated from primary fibroblasts using the RNeasy Mini Kit (Qiagen, Hilden, Germany) according to the manufacturer’s instructions. The concentration and quality of the RNA were determined using the Qubit 4 Fluorometer (Thermo Fisher Scientific, Waltham, MA, USA) and the 2100 Bioanalyzer instrument (Agilent Technologies, Santa Clara, CA, USA). To prepare libraries, 1 μg of RNA was used with the TruSeq Stranded mRNA Library Prep Kit (Illumina, San Diego, CA, USA) according to the manufacturer’s protocol. Then, the NexSeq 500 system (Illumina, San Diego, CA, USA) was used to sequence the mRNA libraries utilizing the highest output mode and paired-end 75 bp read lengths. The RNA-seq data were aligned to the human reference genome GRCh38 by RSEM v1 (https://deweylab.github.io/RSEM/, accessed on 15 March 2021) [70]. RSEM was used to quantify the sequence alignment at the transcript level, and DESeq was used to measure differential gene expression (https://www.bioconductor.org/packages//2.10/bioc/html/DESeq.html, accessed on 15 June 2021) [71].

A functional analysis was performed using IPA (Ingenuity Pathway Analysis) [72] and DAVID Bioinformatics Resources V6.8 (https://david.ncifcrf.gov/, accessed on 1 February 2022) to obtain the role of the gene pool, clinical implication, ontology, and involved metabolic pathways. Moreover, the STRING search tool was used to represent a protein–protein interaction (PPI) network including our target genes (https://string-db.org/, accessed on 16 June 2023) [73].

### 4.6. Validation by Sanger Sequencing 

Selected single nucleotide polymorphism (SNP) variants (SYNJ1, ACOT4, and ACAD9) were validated using Sanger sequencing.

To validate some of the identified variants detected in the exome and identified in the transcriptome, DNA and RNA (cDNA) were amplified for subsequent Sanger sequencing. 

### 4.7. Metabolite Identification

Metabolite extraction was performed on plasma and urine samples from the patient, family members, and healthy controls, with the same age, sex, and weight, according to standard protocols [74]. Liquid chromatography-mass spectrometry (LC-MS) analyses were performed using an UHPLC system (Agilent 1290 Infinity LC System, Waldbronn, Germany) coupled to an LC-QTOF-MS analyzer (6520) (Agilent Technologies, Santa Clara, CA, USA). Gas chromatography-mass spectrometry (GC-MS) analyses were performed using an Agilent Technologies 7890A instrument (Waldbronn, Germany) coupled to a 5975C mass spectrometer (Agilent Technologies, Santa Clara, CA, USA). Capillary electrophoresis-mass spectrometry (CE-MS) analyses were performed on a 7100 CE system coupled to a TOF MS 6224 (Agilent Technologies, Santa Clara, CA, USA). Details on the analytical aspects of this study can be found in [74]. For data analysis, Agilent MassHunter Profinder version B.08.00 was used for the data obtained from the LC-MS and CE-MS analyses, while Agilent MassHunter Unknowns and Agilent MassHunter Qualitative Analysis were used for the data obtained from the GC analyses [74]. The obtained features were filtered by coefficient of variation of the signal (CV) in quality controls. The features were compared by their absolute differences in abundance, expressed as the logarithm of the fold change (log_2_FC). Those characteristics or metabolites that had a log_2_FC < 1.5 were selected and their percentage of change was indicated [74]. Databases such as METLIN, HMDB, LipidMaps, and CEU Mass Mediator were used for the identification of the features found to be significant in the LC-MS and CE-MS analyses, using their MS/MS spectra to support the identification. An in-house built library was used to identify the features obtained in the GC-MS analyses.

## 5. Conclusions

This study helps to elucidate the mechanisms underlying JP, although the cause of JP is multifactorial and not fully understood. For the first time, a novel mutation in the SYNJ1 gene is reported as a potential risk factor for the disease. This feature, together with overexpression of L1CAM, Cdc37, GPX1, and GPX4, reduced CP expression, and alterations in sphingolipid, inositol, and inositol phosphate metabolism, may also play a role in neurodegeneration.

## Figures and Tables

**Table 1:** Transcriptome analysis. The expression differences between the top ten overexpressed genes and the ten genes with lowest expression in the patient compared with the control.

Gene	Difference in Expression	Gene	Difference in Expression
RPS4Y1	15.1559	SP9	−9.5464
L1CAM	14.5911	CD160	−9.5544
GPX1	14.5295	EYS	−9.9120
CDC37	14.3893	AC099850.2	−9.9467
GPX4	14.3795	EPHA3	−10.2126
EMP3	14.3303	C4orf47	−10.4248
S100A16	14.2368	CP	−10.9031
RPS21	14.2270	SFRP2	−10.9031
PSMD8	14.2087	EMCN	−12.9552
EDF1	14.1511	LHX8	−14.2991

**Table 2:** Significant metabolites in samples of plasma from the patient.

Compound	% of Change	
Liquid chromatography		
PC(18:3)	85	Glycerophospholipid, phospholipid, and lipid metabolism, transport, and peroxidation. Fatty acid metabolism.
PC(18:0)	−40	
PC(20:3)	38	
PC(22:4)	−30	
3-Carboxy-4-methyl-5-propyl-2-furanpropionic acid	291	
Arachidonic acid	−32	Lipid metabolism, transport, and peroxidation. Fatty acid metabolism. Arachidonic acid, alpha linolenic acid and linoleic acid metabolism.
Sphingosine 1-Phosphate	−28	
Sphinganine-phosphate	−33	
PE(16:0)	−37	Phospholipid biosynthesis.
PE(18:0)	−40	Phospholipids.
PE(20:3)	−43	
Bilirubin	56	Porphyrin metabolism.
Sphingosine	−33	Sphingolipid metabolism.
Gas chromatography		
Phosphoric acid	48	Arginine, proline, cysteine, glutamate and glutathione, inositol, inositol phosphate, nicotinate and nicotinamide, propanoate, purine, pyrimide, pyruvate, selenoamino acid, and vitamin B6 metabolism. Frutose and mannose degradation. Lactose synthesis. Gluconeogenesis. Urea cycle. Glycolysis. Ammonia recycling. Glycerol phosphate suttle. Warburg effect.
uric acid 1	81	Purine metabolism.
Capillary electrophoresis		
D-Pipecolic acid or N-methyl-L-proline	63	Amino acid metabolism.
N-Acetylneuraminic acid	−58	Amino sugar metabolism.
Myo-inositol	17	Galactose, inositol and inositol phosphate, and phosphatidylinositol phosphate metabolism.
1-Methylnicotinamide	20	Nicotinate and nicotinamide metabolism.
Hydroxymethylbilane	32	Phyrin metabolism.
Uric acid	38	
Guanosine	−39	
3′-AMP	−58	Pyrimidine and tryptophan metabolism. Bile acid biosynthesis..
N-Methylalanine	−50	Sphingolipid metabolism.
O-Phosphoethanolamine	−50	

**Table 3:** Significant metabolites in samples of urine from the patient.

Compound	% of Change	
Liquid chromatography		
2-Hydroxymyristoylcarnitine	121	Lipid metabolism, transport, and peroxidation. Fatty acid metabolism.
3-Methyladipic acid or pimelic acid	inf	
PI(36:1)	125	Glycerophospholipid, phospholipid, and lipid metabolism, transport, and peroxidation. Fatty acid metabolism.
γ-Glutamyl-β-aminopropiononitrile	120	Amino acid metabolism.
Glucose 6-phosphate	390	Gluconeogenesis. Glycogenosis type IB. Glycogenosis type IC. Glycolysis. Galactose, inositol, and inositol phosphate metabolism. Pentose phosphate pathway. Nucleotide sugar metabolism. Starch and sucrose metabolism. Triosephosphate isomerase. Warburg effect.
Capillary electrophoresis		
2-Hydroxy-4-(methylthio) butanoic acid	180	Lipid metabolism, transport, and peroxidation. Fatty acid metabolism.
Dimethylallyl pyrophosphate or isopentenyl pyrophosphate	260	
Nonanoylcarnitine	247	
6-Keto-decanoylcarnitine	252	
Ne,Ne dimethyllysine	218	Amino acid metabolism.
Dipeptide 1	239	
Dipeptide 2	270	
Dipeptide 3	445	
Dipeptide 4	343	
Pipecolic acid or piperidine-2-carboxylic acid	306	Lysine degradation.

## Data Availability

Sequence data have been deposited at the European Genome-phenome Archive (EGA), which is hosted by the EBI and CRG, under accession number EGAS00001007686.

## Supplementary Materials

The following supporting information can be downloaded at: https://www.mdpi.com/article/10.3390/ijms25179754/s1.
