# Supplementary material for: Identification of SYNJ1 in a Complex Case of Juvenile Parkinsonism Using a Multiomics Approach
Source: Int J Mol Sci. 2024 Sep 9;25(17):9754. doi: 10.3390/ijms25179754 (PMC11396201; doi:10.3390/ijms25179754)
Supplement: Supplementary file 1 [file ijms-25-09754-s001.zip › ijms-3137606-supplementary.pdf]

SUPPLEMENTARY

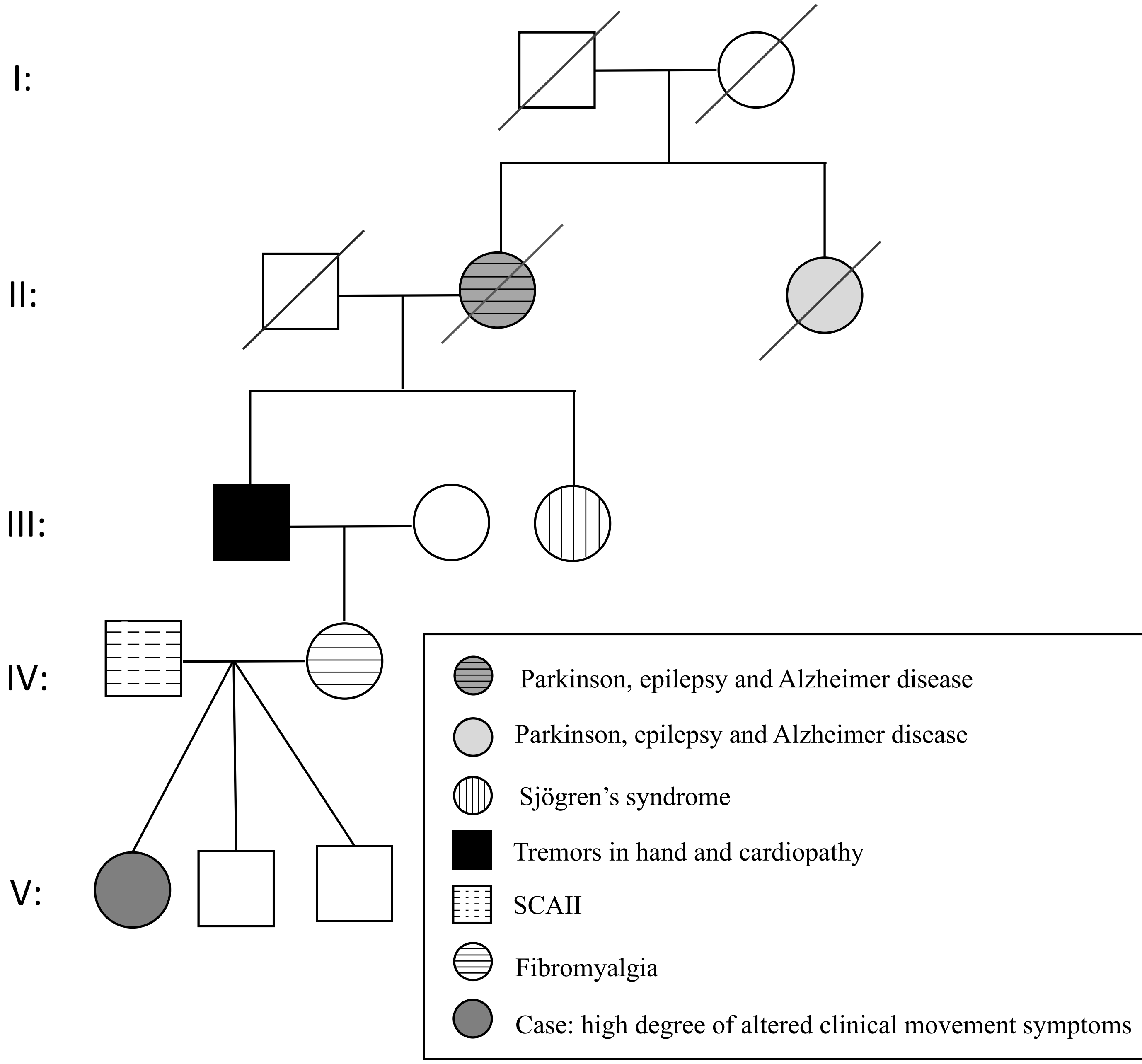

Figure S1. Maternal family history. Square indicates male and circle female, circle and square cross indicates members died. The patient's grandfather (III generations) had tremors in his hand and a cardiomyopathy. In the II generations, a female relative was diagnosed with PD, epilepsy and Alzheimer's disease. Other members of the family present diseases such as fibromyalgia, Sjogren's syndrome, and a cerebral tumor.

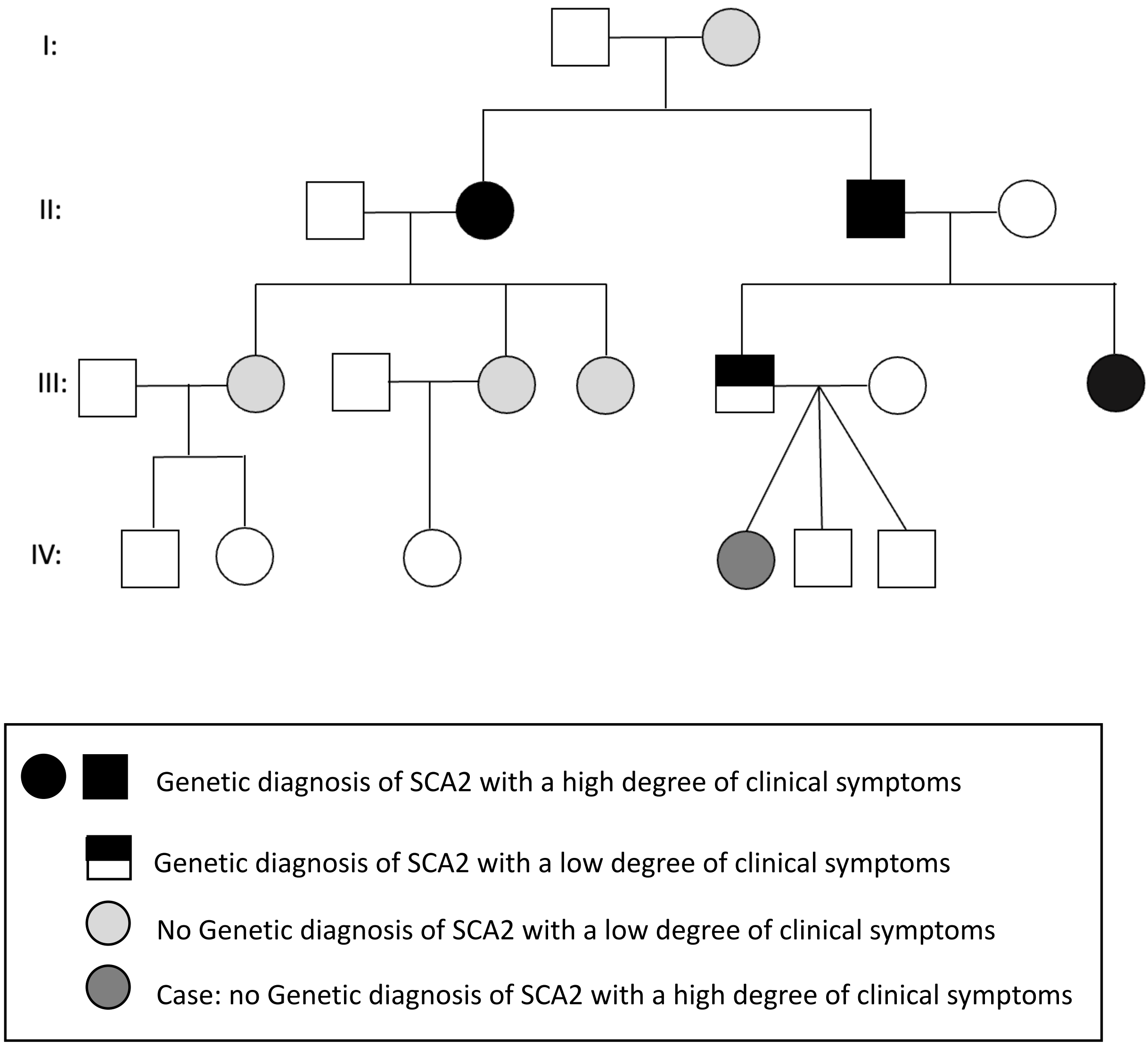

Figure S2. Paternal family SACAII history. The patient's father, IV generations, was diagnosed with spinocerebellar ataxia type 2 (SCA2) with a mild phenotype, and a sister, IV generations, and a female and a male relative of the II generations also present SCA2 with more severe symptoms than the father. Furthermore, three members of the IV generations together with one member of the II generation exhibit an altered phenotype with movement disorders.
